# High prevalence of antibiotic resistance in commensal *Escherichia coli* from healthy human sources in community settings

**DOI:** 10.1038/s41598-021-82693-4

**Published:** 2021-02-09

**Authors:** Emmanuel Nji, Joseph Kazibwe, Thomas Hambridge, Carolyn Alia Joko, Amma Aboagyewa Larbi, Lois Afua Okyerewaa Damptey, Nana Adoma Nkansa-Gyamfi, Cecilia Stålsby Lundborg, La Thi Quynh Lien

**Affiliations:** 1 BioStruct-Africa, Vårby, 143 43 Stockholm, Sweden; 2 Department of Infectious Disease Epidemiology, Imperial College London, London, UK; 3 Department of Public Health, Erasmus MC, University Medical Center Rotterdam, 3015 GD Rotterdam, The Netherlands; 4 Faculty of Science and Engineering, Åbo Akademi University, Turku, Finland; 5 Department of Biochemistry and Biotechnology, College of Science, Kwame Nkrumah University of Science and Technology, PMB, Kumasi, Ghana; 6 The Open University, School of Engineering and Innovation, Walton Hall, Milton Keynes, MK7 6AA UK; 7 Health Systems and Policy (HSP): Improving the Use of Medicines, Department of Global Public Health, Karolinska Institutet, Tomtebodavägen 18A, 17177 Stockholm, Sweden; 8 Department of Pharmaceutical Management and Pharmaco-Economics, Hanoi University of Pharmacy, 13-15 Le Thanh Tong, Hoan Kiem District, Hanoi, 110403 Vietnam

**Keywords:** Bacterial infection, Health policy, Antibiotics, Antimicrobial resistance, Symbiosis

## Abstract

Antibiotic resistance is a global health crisis that requires urgent action to stop its spread. To counteract the spread of antibiotic resistance, we must improve our understanding of the origin and spread of resistant bacteria in both community and healthcare settings. Unfortunately, little attention is being given to contain the spread of antibiotic resistance in community settings (i.e., locations outside of a hospital inpatient, acute care setting, or a hospital clinic setting), despite some studies have consistently reported a high prevalence of antibiotic resistance in the community settings. This study aimed to investigate the prevalence of antibiotic resistance in commensal *Escherichia coli* isolates from healthy humans in community settings in LMICs. Using the Preferred Reporting Items for Systematic Reviews and Meta-Analyses (PRISMA) guidelines, we synthesized studies conducted from 1989 to May 2020. A total of 9363 articles were obtained from the search and prevalence data were extracted from 33 articles and pooled together. This gave a pooled prevalence of antibiotic resistance (top ten antibiotics commonly prescribed in LMICs) in commensal *E. coli* isolates from human sources in community settings in LMICs of: ampicillin (72% of 13,531 isolates, 95% CI: 65–79), cefotaxime (27% of 6700 isolates, 95% CI: 12–44), chloramphenicol (45% of 7012 isolates, 95% CI: 35–53), ciprofloxacin (17% of 10,618 isolates, 95% CI: 11–25), co-trimoxazole (63% of 10,561 isolates, 95% CI: 52–73), nalidixic acid (30% of 9819 isolates, 95% CI: 21–40), oxytetracycline (78% of 1451 isolates, 95% CI: 65–88), streptomycin (58% of 3831 isolates, 95% CI: 44–72), tetracycline (67% of 11,847 isolates, 95% CI: 59–74), and trimethoprim (67% of 3265 isolates, 95% CI: 59–75). Here, we provided an appraisal of the evidence of the high prevalence of antibiotic resistance by commensal *E. coli* in community settings in LMICs. Our findings will have important ramifications for public health policy design to contain the spread of antibiotic resistance in community settings. Indeed, commensal *E. coli* is the main reservoir for spreading antibiotic resistance to other pathogenic enteric bacteria via mobile genetic elements.

## Introduction

Antibiotic resistance (ABR) is currently identified as one of the biggest threats to not only global health but also to food security and development^1^. Resistance occurs when the antibiotics (medicines used to prevent and treat bacterial infections) are no longer effective at inhibiting the growth of the bacteria^1^. There is a growing increase of resistance by bacteria to antibiotics^2–10^ with the World Health Organization (WHO) through its Global Antimicrobial Surveillance System (GLASS) report revealing that there are high levels of antibiotic resistance in both low- and high-income countries^11^. In fact, the European Centre for Disease Prevention and Control (ECDC) reported that 25,000 people died of diseases caused by antibiotic-resistant bacteria in 2007, which is over half the number caused by road traffic accidents in the same countries^12^. In 2015, this number increased to about 33,000 deaths resulting from an estimated 671,689 infections of selected antibiotic-resistant bacteria leading to 874,541 total disability-adjusted life-years (DALYs)^13^. This indicates that the burden in the European Union and European Economic Area is on the rise. Likewise, the World Health Organization (WHO) predicted that by 2050, the number of people who will die due to antibiotic resistance would increase from 700,000 to about 10 million per year globally^14^. As a result of antibiotic resistance, more than 2.8 million people are infected, and more than 35,000 die each year in the USA^15,16^.

The burden caused by antibiotic resistance is greater in low- and middle-income countries (LMICs) whose health care systems are poor and lack tools to perform rapid diagnosis of the numerous neglected infectious diseases^17^. In the community settings in LMICs, high prevalence of multidrug-, extensive drug-, and pan drug-resistant commensal *Escherichia coli* isolated from healthy humans has been reported^18^. Thus, greater efforts should be placed in LMICs to contain the spread of antibiotic-resistant *E. coli*, especially with its relaxed antibiotics prescription policies. Antibiotic resistance has led to an increase in poverty in LMICs^16^, and antibiotics misuse is associated with the carriage of resistant commensal *E. coli* from healthy children in community settings worldwide^19,20^. In high-income countries, stringent antibiotic prescription policies are in place to reverse the course of antibiotic resistance^21^.

**Figure 1:**
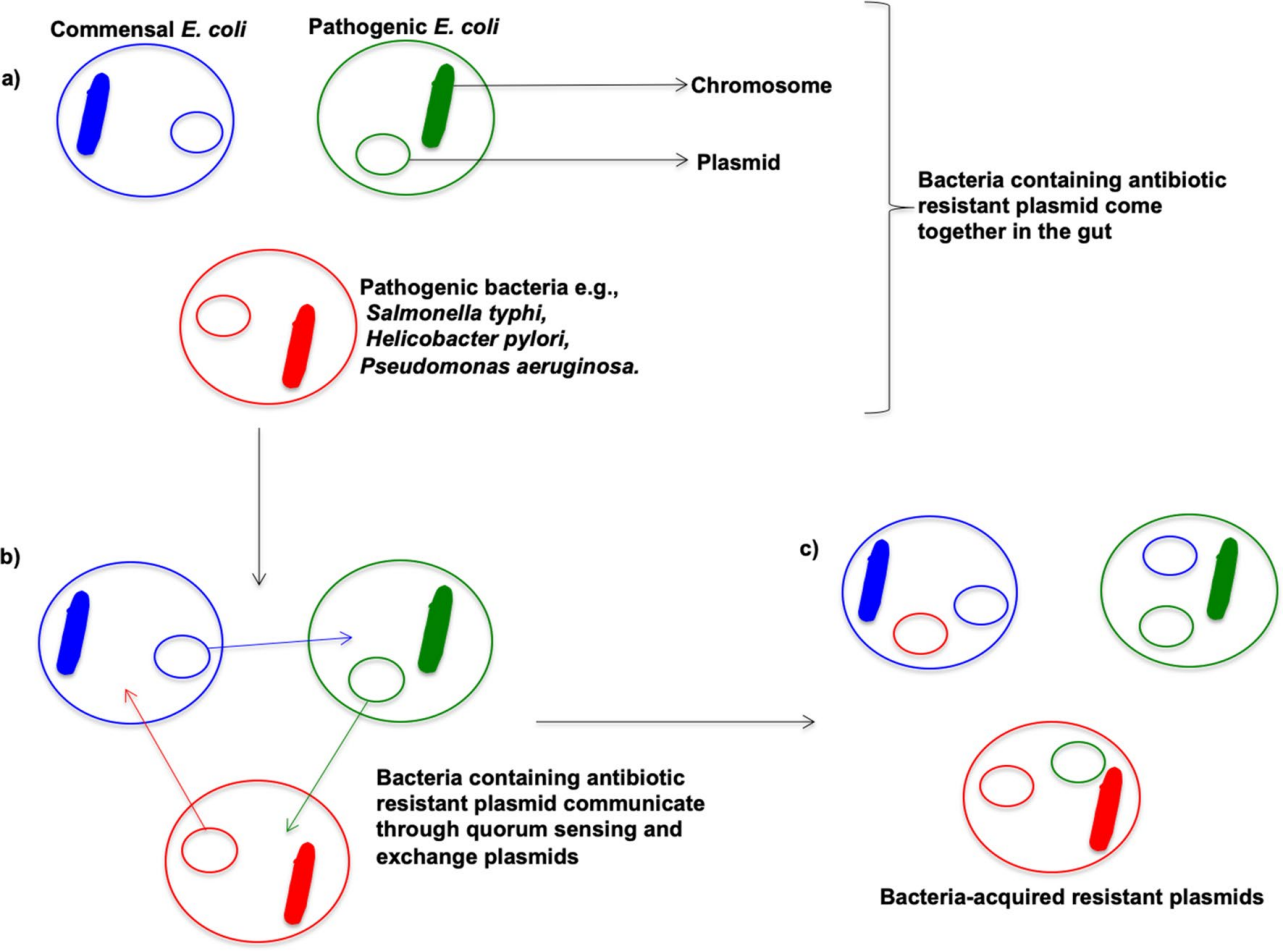
Transfer of resistance between bacteria through plasmid exchange. **(a)** Commensal *E. coli,* pathogenic *E. coli* and other pathogenic bacteria come together in the gut, **(b)** Bacteria attached and exchanged plasmids conferring antibiotic resistance. **(c)** Bacteria-acquired resistant plasmids.

Commensal *Escherichia coli* is a gram-negative bacterium located in the gut of humans, animals, birds, and also exists in the environment^22^. It is a pathogen on the WHO global critical pathogen priority list for research, discovery, and new antibiotics development^23^. When we ingest antibiotics for the treatment of bacterial infections, the commensal *E. coli* is exposed to these antibiotics and can develop resistance to these antibiotics through natural selection^24^. Indeed, commensal *E. coli* is one of the major reservoirs for the transmission of antibiotic resistance to other pathogenic bacteria through plasmid exchange, for example (Fig. 1)^25–32^. Humans can be exposed to viable commensal antibiotic-resistant *E. coli* by contact with livestock or a contaminated natural environment and by inadequately cooked food or cross-contamination^33–35^.

Many studies have shown a high prevalence of resistance to antibiotics by pathogenic and commensal bacteria in healthcare settings^8^. However, when comparing these studies to those conducted in community settings there is a large discrepancy, especially in LMICs^36^. This is as a result of the fact that little attention is given at the community level to contain the global antibiotic resistance crises; despite some studies that compare the prevalence of resistance to antibiotics in both communities and hospitals all showed consistently high values with no significant difference^8,22,37–40^. Thus, similar attention should be given to contain the cause of resistance to antibiotics by bacteria in communities, as is the case in hospitals. If this situation is not addressed, many of the gains in modern medicine will be lost and the commitment to achieve universal health coverage by world leaders will be in vain^7,41^. In this paper, we aim to provide an appraisal of the evidence of the high prevalence of antibiotic resistance by commensal *E. coli* to commonly prescribed antibiotics in community settings (i.e., locations outside the hospital such as homes and schools) in LMICs to bring to light the extent of the problem and inform interventions targeted at controlling and preventing antibiotic resistance. Indeed, a multitude of knowledge, attitudes, and practices (KAP), education, and community engagement interventions exist in community settings in low- and middle-income countries (LMICs), yet data to support and justify their set-up are often lacking. Thus, our data should prove useful to support the course for the fight against antibiotic resistance by researchers, community pharmacists, public health policymakers, advocacy groups, farmers, among others. A collective approach involving every country to fight antibiotic resistance is crucial to reduce the mortality, morbidity, associated health and healthcare costs, and the spread of resistant bacteria^10,42^.

## Results

### Literature search

**Figure 2:**
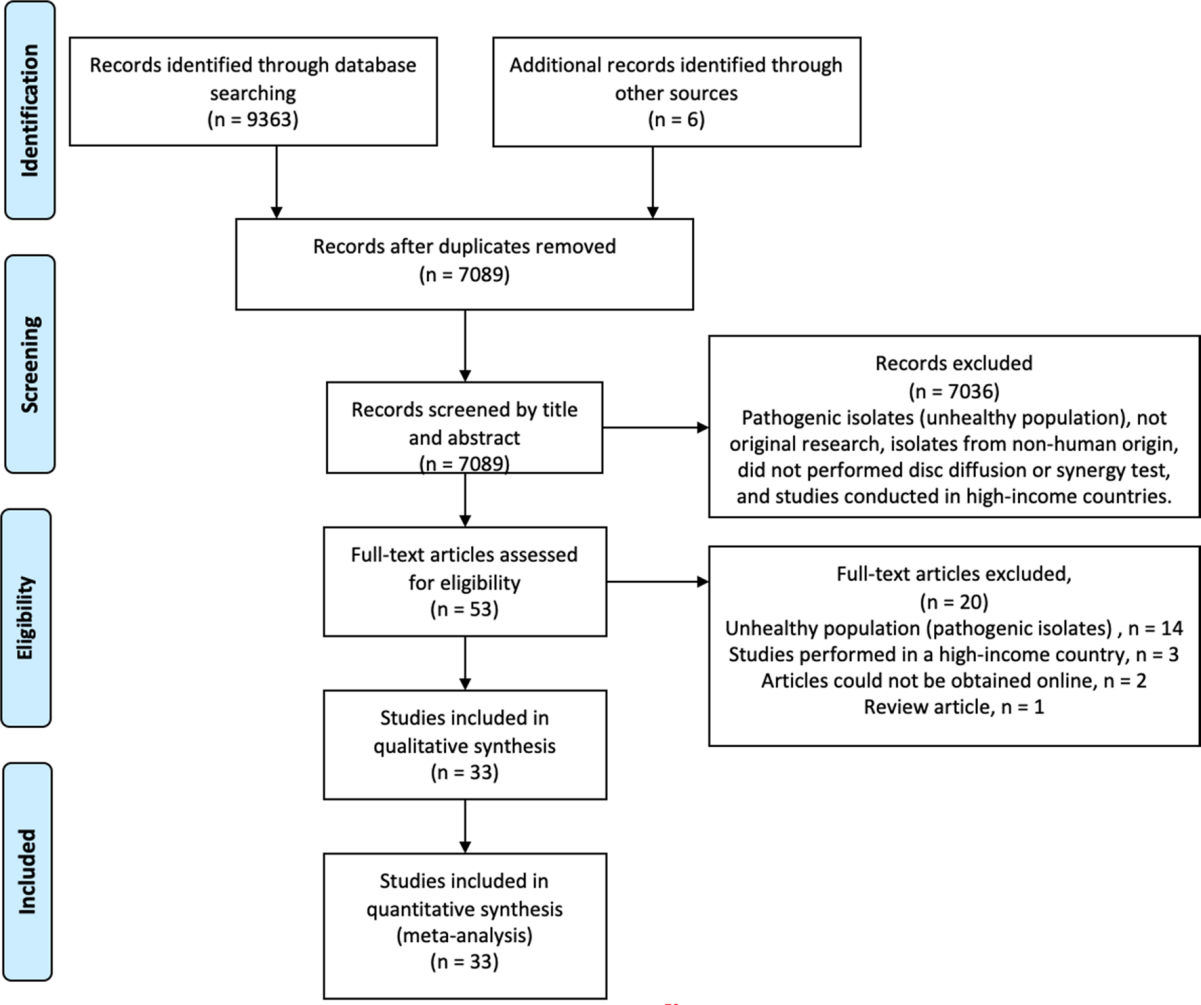
Flow diagram of the literature search strategy^73^. Additional records were identified from the reference lists of some of the included studies.

A total of 9363 articles were obtained from the search (PubMed = 3634, EMBASE + MEDLINE = 2103, Web of Science = 3046, CINAHL = 290 and Cochrane Library = 289). Out of the 9363 articles, 2280 duplicates were removed using EndNote X8. We screened 7089 articles to identify article hits that met our inclusion criteria (Fig. 2). We performed a full-text screening of 53 studies and data were extracted from 33 articles^8,28,29,43–72^. A total of 20 articles in which the isolates were pathogenic (14), were collected in a high-income country (3), could not be obtained online (2), or review articles (1) were excluded for data extraction.

### Study location, strength of evidence, and study type

**Figure 3:**
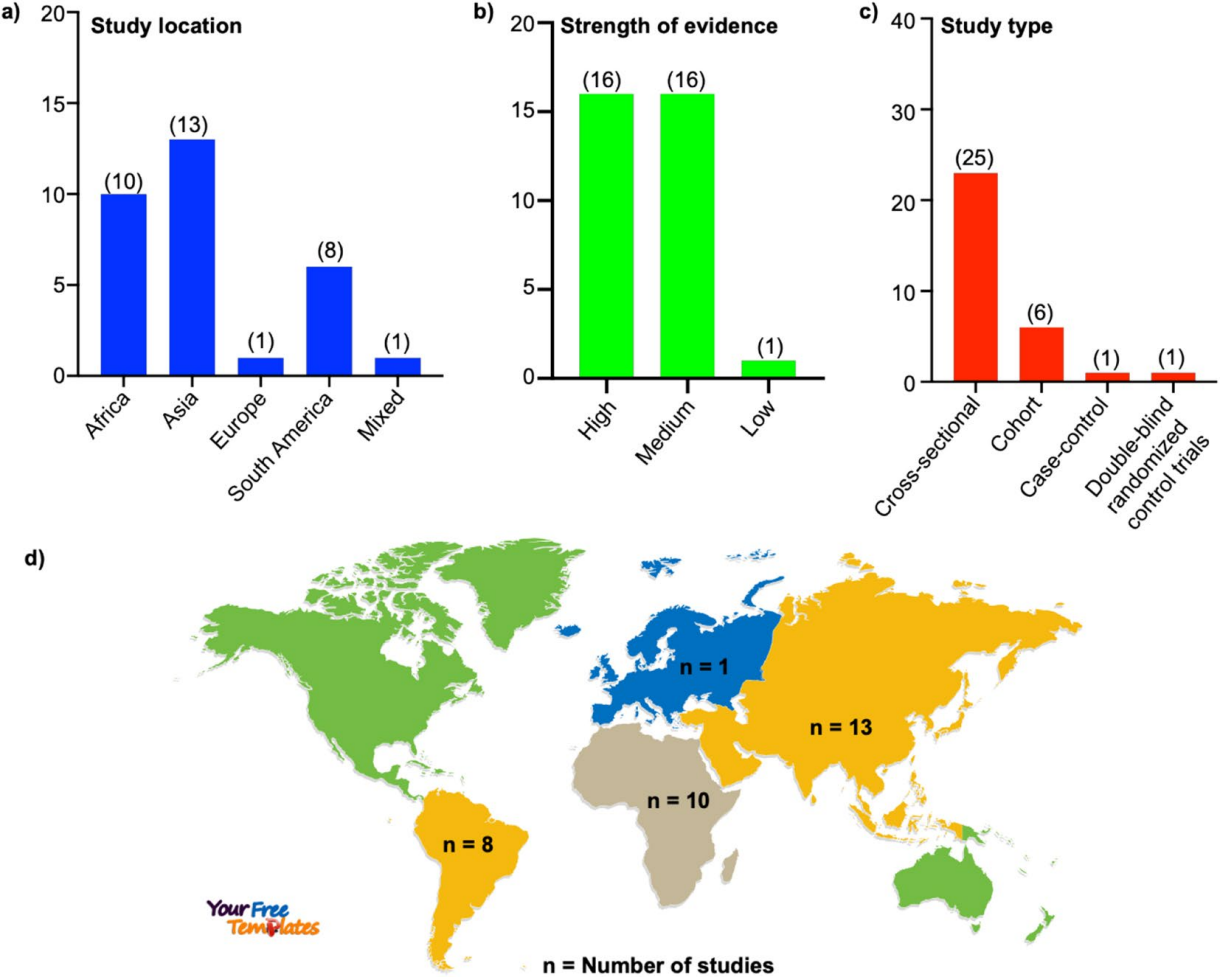
Characteristics of the included studies. **(a)** Location, **(b)** quality of the evidence, and **(c)** type of studies. **(d)** World map showing the number of studies by continents. The world map was obtained from (https://yourfreetemplates.com/) on 17th December 2020.

Out of the 33 included studies, 10 were from Africa, 13 from Asia, 1 from Europe, 8 from South America, and 1 from multiple locations (Fig. 3a,d). The quality of the evidence was assessed as described previously^74,75^. Of the 33 studies, 16 were marked as high quality, 16 as a medium, and 1 as low quality (Fig. 3b). These included studies were cross-sectional (25), cohort (6), case–control (1), and double-blind randomized control trials (1) (Fig. 3c).

### Age, sample size, and gender

The included studies comprise of about 7755 health individuals in community settings from 0 to 77 years of age from 1989 to 2019. One limitation of this study is that some articles did not record the number of males and females, thus it was challenging to determine the ratio of males to females. However, we calculated the number of males and females using the studies that recorded the numbers. Of these studies, 2443 were males and 2010 were females. Thus, assuming a total population of 7755, we extrapolated the number of males to be 4255, and females 3500.

### High prevalence of antibiotic resistance in the community settings will potentially impact health policy

**Figure 4:**
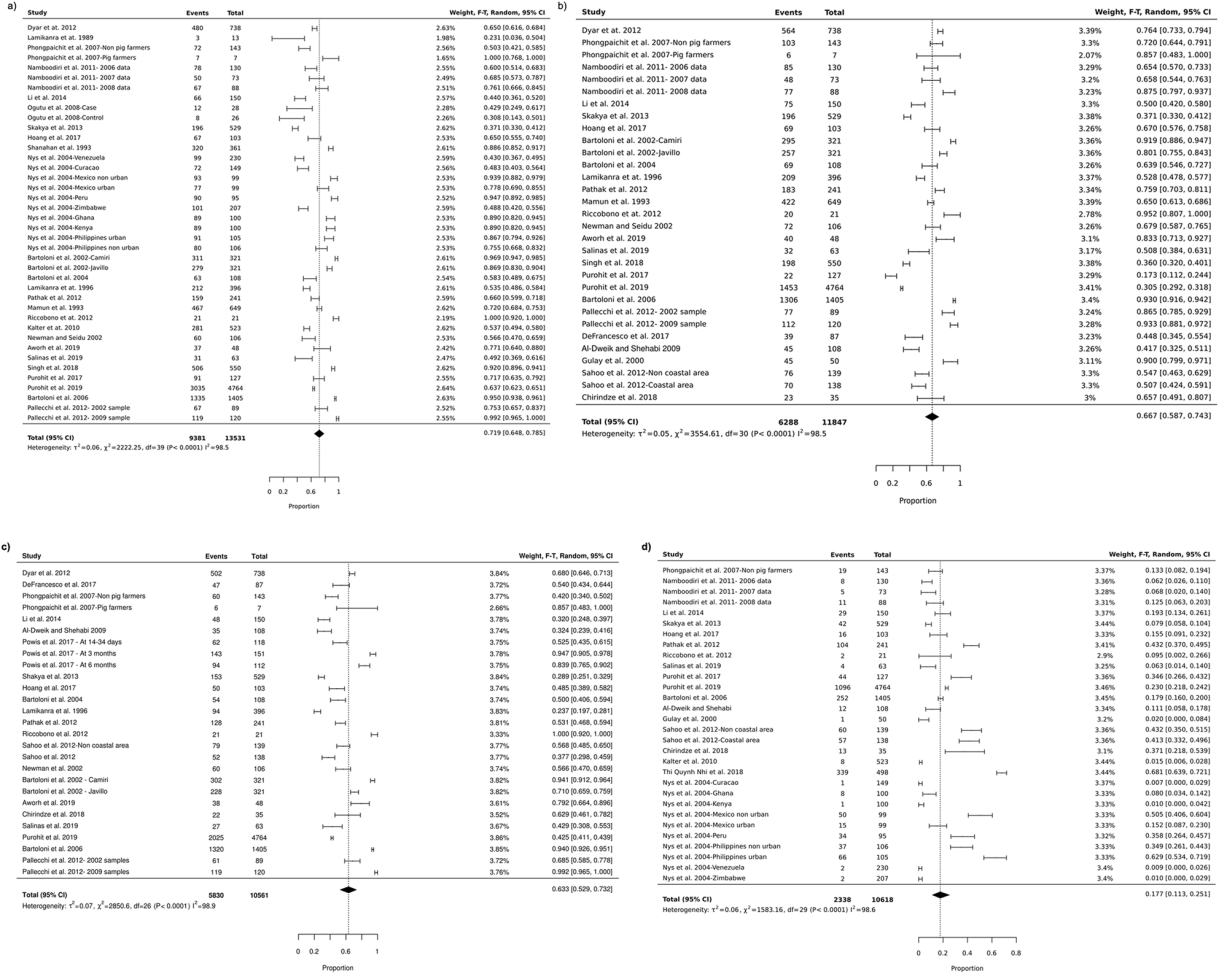
Forest plots showing the prevalence of antibiotic resistance in commensal *E. coli* isolated from human sources in community settings in low- and middle-income countries. **(a)** Cell wall synthesis inhibitor (ampicillin), **(b)** protein synthesis inhibitor (tetracycline), **(c)** folate synthesis inhibitor (co-trimoxazole), and **(d)** nucleic acid synthesis inhibitor (ciprofloxacin).

The pooled prevalence of commensal *E. coli* isolated from healthy individuals in community settings in LMICs for the different antibiotics are summarized in Table 1, Fig. 4, and Supplementary Fig. 1. A high prevalence was seen for some of the commonly prescribed antibiotics in these countries like ampicillin (72%, 95% CI: 65–79), cefotaxime (27%, 95% CI: 12–44), chloramphenicol (45%, 95% CI: 35–53), ciprofloxacin (17%, 95% CI: 11–25), co-trimoxazole (63%, 95% CI: 52–73, nalidixic acid (30%, 95% CI: 21–40), oxytetracycline (78%, 95% CI: 65–88), streptomycin (58%, 95% CI: 44–72), tetracycline (67%, 95% CI: 59–74), and trimethoprim (67%, 95% CI: 59–75). These findings will be very useful for evidence-based health policy design aimed at combating the spread of antibiotic resistance in the community.

**Table 1:** Prevalence of antibiotic resistance in commensal *E. coli* isolated from human sources in community settings in low- and middle-income countries.

Antibiotics	Mechanism of inhibition	Study number	Total number of isolates	Number of resistant isolates	Pooled prevalence (%)	Lower bound 95% CI	Upper bound 95% CI	I^2^ (%)	Quality of the evidence (study number)	Reference
Ampicillin	Cell wall synthesis	25	13,531	9381	72	65	79	99	High (13), medium (11), low (1)	^8,28,29,43,44,46,47,49,50,53,55,58–66,68–72^
Cefotaxime	Cell wall synthesis	10	6700	3493	27	12	44	99	High (3), medium (7), low (0)	^28,29,43,47,48,53,55,57,58,60^
Chloramphenicol	Protein synthesis	18	7012	3343	45	35	53	99	High (11), medium (6), low (1)	^8,28,29,43,44,47–49,53,59,60,62–64,66,68–70^
Ciprofloxacin	Nucleic acid synthesis	19	10,618	2338	17	11	25	99	High (11), medium (8), low (0)	^26,37,38,41–43,45,47–52,55,58–60,65,66^
Co-trimoxazole	Folate synthesis	20	10,561	5830	63	52	73	98	High (10), medium (10), low (0)	^8,28,29,43,45,47,49–53,55–57,59–61,63,70,72^
Nalidixic acid	Nucleic acid synthesis	21	9819	3960	30	21	40	99	High (10), medium (10), low (1)	^8,28,29,43,46–49,51,55,57–59,61,63,64,66,68–71^
Oxytetracycline	Protein synthesis	2	1451	1047	78	65	88	96	High (0), medium (2), low (0)	^44,67^
Streptomycin	Protein synthesis	13	3831	2610	58	44	72	99	High (9), medium (3), low (1)	^29,47,48,53,61–64,66,69,70,72^
Tetracycline	Protein synthesis	25	11,847	6288	67	59	74	99	High (15), medium (10), low (0)	^8,28,29,43,45,47–51,53,55–64,66,70–72^
Trimethoprim	Folate synthesis	9	3265	1854	67	59	75	98	High (3), medium (5), low (1)	^28,44,46,48,64,66–69^

### Prevalence of antibiotic resistance data of a plethora of antibiotics will potentially impact antibiotic stewardship programs

In this study, the prevalence of resistance was collected for a range of diverse antibiotics and mechanisms of actions such as, inhibition of protein, nucleic acid, folic acid, or cell wall synthesis. For some antibiotics, the resistance was very high, others were emerging, and for some it was low (Table 1 and Supplementary Table 4). This evidence should prove useful to inform antibiotic stewardship programs.

### Investigating the source of heterogeneity

The results show a high I^2^ value, which indicates considerable heterogeneity. Funnel plots and Egger’s regression test was used to explore the sources of heterogeneity. The funnel plots for all antibiotics were asymmetrical (Supplementary Fig. 2), thus indicating a possibility of publication bias. We further investigate the existence of publication bias per antibiotic using Egger’s regression test (Supplementary Table 5). Most of the antibiotics (ampicillin, cefotaxime, co-trimoxazole, nalidixic acid, streptomycin, and trimethoprim) did not exhibit publication bias. The antibiotics that exhibited publication bias were chloramphenicol, ciprofloxacin, oxytetracycline, and tetracycline. On further stratification of each antibiotic prevalence, according to continents (Africa, South America, and Asia), there was a reduced likelihood of publication bias based on visual examination of the funnel plot.

## Discussion

Antibiotic resistance (ABR) is a serious global health threat that needs to be addressed urgently^2–8^. ABR's impact is particularly greater in low- and middle-income countries (LMICs), which bear the highest-burden and subsequently suffer the most from this problem, primarily because their healthcare systems lack the resources needed to contain or to treat challenging infectious diseases caused by drug-resistant bacteria^17^. Indeed, it has led to an increase in poverty in LMICs^16^. While there has been a general increase in multidrug-resistant pathogenic bacteria in community settings^77^ recent evidence suggests that the prevalence of multidrug-resistant commensal *Escherichia coli* isolated from healthy individuals is particularly high in LMICs^18^. In this study, we synthesized a total of 33 articles to obtain a pooled prevalence of ABR in the top ten antibiotics commonly prescribed in community settings (i.e., locations outside of a hospital, such as schools and homes) in LMICs.

There are several factors that contribute to ABR^78^, with a complicated inter-relationship that spans across different sectors outside of healthcare alone, such as agriculture and industry. Among the main factors identified leading to resistance in commensal *E. coli* in LMICs are overcrowding, poverty, socioecological behaviours, food and supply chain safety issues, highly contaminated waste effluents and inadequate surveillance systems^79^. Nevertheless, the primary driver of multidrug-resistance in LMICs has been misuse and over-prescription of antibiotics^80^. Commensal *E. coli* are typically present in the guts of humans, animals, birds, as well as in the environment, and can develop resistance to antibacterial agents through natural selection when ingested for the treatment of bacterial infections^22,24^.

This review revealed a high prevalence of antibiotic resistance in commensal *E. coli* to the most prescribed antibiotics in LMICs. Moreover, this was a consistent finding across several classes of antibiotics with different mechanisms of action. For instance, the pooled prevalence of antibiotic resistance for the β-lactam antibiotic ampicillin was 72%, 95% CI: 65–79, while for trimethoprim, a folic acid synthesis inhibitor, the pooled prevalence was 67%, 95% CI: 59–75. Similar observations were reported in a recent systematic review investigating ABR in *E. coli* strains isolated from humans, animals, food, and the environment in several middle- and high-income countries. The authors presented high rates of resistance against a range of antibiotics found in *E. coli* isolates, although the pooled prevalence was generally lower than in the isolates from healthy individuals from LMICs presented here^81^. For ciprofloxacin, the pooled prevalence from our study was 17%, 95% CI: 11–25, which is in the range of the resistance seen for treating *E. coli* associated urinary tract infection (8% to 65%)^11^, and other *E. coli* isolated from farmed minks in Zhucheng, China^76^. Likewise, for cotrimoxazole, our data (63%, 95% CI, 52–73) agrees with another study carried out in Zimbabwe, where the prevalence was 68% for Gram-negative bacillli^82^. The main limitation of our study is the fact that the heterogeneity between studies was very high (Table 1, Fig. 4). We performed an additional statistical analysis stratified by continent. The goal was to solve the heterogeneity issue, however, there was no significant difference between the pooled prevalence of antibiotic resistance values between continents. The high heterogeneity between studies could stem from the different factors associated with the carriage resistant commensal *E. coli* in LMICs highlighted in the discussion section above. Since I^2^ statistics test for heterogeneity can be misleading during meta-analysis of observational studies^83,84^, we performed an alternative assessment of the strength of evidence of the different studies (supplementary table 3)^74,75^. The included studies utilized disc diffusion or synergy test to investigate the expression of resistant genes in the presence of antibiotics. However, those that employed an additional method, such as PCR, plasmid transfer assay, nucleic acid identification, mass spectrometry, to validate the presence of genes conferring resistance to a particular antibiotic were graded as high.

Our study is in line with the WHO’s Global action plan on antimicrobial resistance, which calls for improved awareness of the problems arising from antibiotic resistance as stated in one of the five strategic objectives^85^. Indeed, raising awareness can be facilitated, for example, through the making of participatory videos^86^. As expected, in this study, resistance was commonly detected from stool samples collected from healthy volunteers in community settings. Our findings have important ramifications for public health policies and antibiotic resistance stewardship through a one-health approach for the fight against ABR. In some of the studies screened, factors such as previous antibiotic use^19,55,59,65^, geographical location^8,57^, age^8,59^, socioeconomic status^43,55,57^, and exposure to animals^49,65^ were highlighted as being associated with a high prevalence of resistance. The situation is even more pressing with the emergence of global pandemics such as the coronavirus disease 2019 (COVID19) caused by the Severe Acute Respiratory Syndrome Coronavirus 2 (SARS-CoV-2)^87^. Prior to the availability of approved vaccines, different medicines have been tested randomly in clinical trials to find a cure for this deadly pandemic^87–94^. Since viral infections are often associated with bacterial infections^95,96^, the antibiotic azithromycin in combination with the antimalarial drug hydroxychloroquine was proposed as an option for the treatment of COVID19 patients^97^. Thus, we may see a post-COVID19 global health crisis with a surge in antibiotic resistance leading to many deaths^97^.

## Conclusion

This study provides further evidence of the high prevalence of antibiotic resistant commensal *E. coli* from healthy human sources in community settings in LMICs. These findings should encourage health researchers, medical professionals, advocacy groups, and health policymakers to work together to develop appropriate interventions to counteract this growing global health threat. We recommend that the strategies that have been implemented in healthcare settings to contain the spread of resistance, such as surveillance, raising awareness, improve sanitation and hygiene, rapid diagnosis of diseases, and stringent prescription policies should also be urgently implemented in the communities to curb antibiotic resistance.

## Methods

### Design

A systematic approach was used to retrieve and synthesize studies that met our inclusion criteria following PRISMA guidelines^73^.

### Type of studies

The types of studies included in the systematic review are those in which the main outcome was the prevalence of antibiotic resistance in commensal *Escherichia coli*: cross-sectional, case–control, cohort studies, and randomized control trials.

### Type of participants (study population)

This review included studies concerning the general healthy populations in community settings in LMICs.

### Outcome of interest

- The primary outcome was prevalence of resistance to antibiotic by commensal *E. coli* in community settings in LMICs.
- The secondary outcomes were: odds ratio, risk ratio, rate, 95% confidence interval, and p-value.

### Other inclusion and exclusion criteria

Articles written in English language were included from 1989 to May 2020 in LMICs. Articles containing studies conducted in a country that was classified as a LMIC before transforming into a high-income country according to the World Bank definition were also included. The included studies must investigate the resistance of commensal *E. coli* on either solid or liquid growth media in the presence of antibiotics.

### Essential data necessary for inclusion

Studies were eligible for inclusion if they reported at least one of the primary or secondary outcomes listed in the *outcome of interest* section above.

### Data sources

Published data from PubMed, EMBASE, MEDLINE, Web of Science, CINAHL and Cochrane Library, reference lists of selected studies and unpublished data such as abstracts from Conference proceedings; dissertations and theses were the data sources.

### Systematic search strategy

A literature search was performed on the 10th of March 2018 and was updated on the 18th of May 2020. Briefly, we searched PubMed, EMBASE, MEDLINE, Web of Science, CINAHL, and Cochrane Library using MeSH terms for PubMed and the comparable terms for the other databases. The search terms were “*E. coli* OR *Escherichia coli* OR Enterobacteriaceae” AND "antibiotic resistance OR antimicrobial resistance OR drug resistance” AND "prevalence OR incidence OR morbidity OR odd ratio OR risk ratio OR confidence interval OR p-value OR rate". For PubMed, EMBASE, and MEDLINE, studies performed in humans were selected using the Species filters. While for Web of Science and Cochrane Library additional search words were added to select for species (human* OR infant* OR child* OR adolescen* OR male* OR female OR age OR adult*) since there was no sorting filter for species. For CINAHL, no selection for species was performed. Search words were designed from the different categories in the PICO (Population, Intervention, Comparison, and Outcome) format. Details of the search terms used are summarized in Supplementary Table 1. The articles obtained from the search were exported to EndNote for duplicate removal. The unique hits were further exported to Rayyan QCRI website for screening and data extraction^98^. An initial screening was performed by title and abstract, followed by full article text.

### Data extraction and quality assessment

A two-step process was followed involving screening of titles and abstracts to identify relevant articles, which was followed by full-text reading of the relevant articles. A total of 53 full-text articles were screened, and the data extracted and recorded on an excel spreadsheet by five researchers. To exclude selection bias, a sixth researcher was available to solve the disagreements that arose during the data extraction. The parameters that were extracted are listed in Supplementary Table 2. The quality of evidence in the included studies was assessed as described before^72,73^ (Supplementary Table 3). In brief, studies that utilized disc diffusion or synergy test in combination with one of the following biochemical test such as, polymerase chain reaction (PCR), plasmid transfer assay (PTA), pulsed-field gel electrophoresis (PFGE), nucleic acid sequencing, and mass spectrometry to detect the presence of resistant genes was graded as high. Furthermore, studies which use only disc diffusion of synergy test with a sample size less than 15 were classified as medium. Lastly, studies which sample size of below 15 and utilized only disc diffusion or synergy test were classified as low.

### Statistical analysis

Statistical analysis was performed in Stata^99^ and JBI SUMARI^100^. The prevalence of antibiotic resistance of commensal *E. coli* was defined as the proportion of the isolates in a specific study that were found to be resistant to a given antibiotics presented as a percentage. The pooled prevalence was calculated using the metaprop command in Stata^101^. *Metaprop* pools proportions and presents a weighted sub-group and overall pooled estimates with inverse-variance weights obtained from a random-effects model. In this case, it involved a meta-analysis of the prevalence values of the individual publications weighted on sample size while accounting for potential heterogeneity between studies. For JBI SUMARI, proportional meta-analysis was calculated using the random-effects model of Freeman-Tukey transformation. Besides, a forest plot was constructed for each of the top ten most reported antibiotics in our study.

The source of heterogeneity was explored by stratifying the pooled prevalence by year of sample analysis (study period), geographical location (continents), and type of antibiotic. Funnel plots of overall effect size were run to determine the existence of publication bias by visual inspection. Also, the Egger’s test was used to assess the occurrence of small size effect. The level of significance was maintained at 0.05.

## Supplementary Information

Supplementary Information.

## Data Availability

Data related to the manuscript is available upon request to corresponding author.
