# Supplementary material for: High prevalence of antibiotic resistance in commensal Escherichia coli from healthy human sources in community settings
Source: Sci Rep. 2021 Feb 9;11:3372. doi: 10.1038/s41598-021-82693-4 (PMC7873077; doi:10.1038/s41598-021-82693-4)
Supplement: Supplementary file 1 — Supplementary Information. [file 41598_2021_82693_MOESM1_ESM.pdf]

High prevalence of antibiotic resistance in commensal *Escherichia coli* from healthy human sources in community settings

Emmanuel Nji[1,*], Joseph Kazibwe[2], Thomas Hambridge[3], Carolyn Alia Joko[1,4], Amma Aboagyewa Larbi[1,5], Lois Afua Okyerewaa Damptey[6], Nana Adoma Nkansa-Gyamfi[1], Cecilia Stålsby Lundborg[7], La Thi Quynh Lien[7,8].

[1]BioStruct-Africa, Vårby, SE-143 43, Stockholm, Sweden.
[2]Department of Infectious Disease Epidemiology, Imperial College London, UK.
[3]Department of Public Health, Erasmus MC, University Medical Center Rotterdam, 3015 GD Rotterdam, Netherlands.
[4]Faculty of Science and Engineering, Åbo Akademi University, Turku, Finland.
[5]Department of Biochemistry and Biotechnology, College of Science, Kwame Nkrumah University of Science and Technology, PMB, Kumasi, Ghana.
[6]The Open University, School of Engineering and Innovation, Walton Hall, Milton Keynes, MK7 6AA, UK.
[7]Health Systems and Policy (HSP): Improving the Use of Medicines, Department of Global Public Health, Karolinska Institutet, Tomtebodavägen 18A, 17177 Stockholm, Sweden.
[8]Department of Pharmaceutical Management and Pharmaco-Economics, Hanoi University of Pharmacy, 13-15 Le Thanh Tong, Hoan Kiem District, 110403 Hanoi, Vietnam.
[*]Correspondence: Email: emmanuel.nji@biostructafrica.org, Tel: 0046720185443

Supplementary Table 1. Details of the search strategy used for the different databases.

Database	Search words	Articles
PubMed	((((((Escherichia coli[MeSH Terms]) OR E. coli[Title/Abstract]) OR Enterobacteriaceae[Title/Abstract])) AND (((((((((Prevalence[MeSH Terms]) OR prevalence[Title/Abstract]) OR incidence[Title/Abstract]) OR morbidity[Title/Abstract]) OR odd ratio[Title/Abstract]) OR confidence interval[Title/Abstract]) OR risk ratio[Title/Abstract]) OR rate[Title/Abstract]) OR p value[Title/Abstract])) AND (((((((antibiotic resistance[MeSH Terms]) OR antibiotic resistance[Title/Abstract]) OR antimicrobial resistance[MeSH Terms]) OR antimicrobial resistance[Title/Abstract]) OR "drug resistance"[MeSH Terms]) OR "drug resistance"[Title/Abstract]) AND English[lang]))	3634
EMBASE + MEDLINE	(*'escherichia coli'*:ab,ti OR *'e. coli'*:ab,ti 'enterobacteriaceae':ab,ti) AND ('antibiotic resistance':ab,ti OR 'antimicrobial resistance':ab,ti OR 'drug resistance':ab,ti) AND ('prevalence':ab,ti OR 'incidence':ab,ti OR 'morbidity':ab,ti OR 'odd ratio':ab,ti OR'risk ratio':ab,ti OR 'confidence interval':ab,ti OR 'p value':ab,ti OR 'rate':ab,ti)	2107
WEB OF SCIENCE	(*E. coli* OR *Escherichia coli* OR Enterobacteriaceae) *AND* **TOPIC:** (Antibiotic resistance OR antimicrobial resistance OR drug resistance) *AND* **TOPIC:** (Prevalence OR Incidence OR Morbidity OR Odd ratio OR Risk ratio OR Confidence interval OR P value OR rate) *AND* **TOPIC:** (HUMAN* OR INFANT* OR CHILD* OR ADOLESCEN* OR MALE* OR FEMALE)	3616
CINAHL	TI (*E. coli* OR *Escherichia coli* OR Enterobacteriaceae) OR AB (*E. coli* OR *Escherichia coli* OR Enterobacteriaceae) AND TI (Antibiotic resistance OR antimicrobial resistance OR drug resistance) OR AB (Antibiotic resistance OR antimicrobial resistance OR drug resistance) AND TI (Prevalence OR Incidence OR Morbidity OR Odd ratio OR Risk ratio OR Confidence interval OR P value OR rate) OR AB (Prevalence OR Incidence OR Morbidity OR Odd ratio OR Risk ratio OR Confidence interval OR P value OR rate).	290
COCHRANE LIBRARY	*E. coli* OR *Escherichia coli* OR Enterobacteriaceae in Title, Abstract, Keywords and Antibiotic resistance OR antimicrobial resistance OR drug resistance in Title, Abstract, Keywords and Prevalence OR Incidence OR Morbidity OR Odd ratio OR Risk ratio OR Confidence interval OR P value OR rate in Title, Abstract, Keywords and HUMAN*	289

Supplementary Table 2. Key information extracted from the studies.

**General information**	**Study characteristics**	**Participant characteristics**	**Measurement tools/methods used in the study**	**Outcome of interest**
First author	Study design	Age	Method used to control bias	Prevalence of antibiotic resistance (%)
Year of publication	Study duration	Gender	Method used to detect *E. coli* species	Factors associated with the carriage of resistance (OR, RR, rate, 95% CI, p-value
Journal	Number of isolates	Geographical location	Methods used to detect resistance	
Country of origin	Sample size	Ethnicity		
PubMed ID		Socio-economic status		

Supplementary Table 3. Reference scale used for accessing the quality of evidence of the selected studies.[1,2] **Polymerase chain reaction (PCR), plasmid transfer assay (PTA), pulsed field gel electrophoresis (PFGE), nucleic acid sequencing, and mass spectrometry, detects the presence of resistant genes while disc diffusion/synergy test determines the expression of resistant genes.**

**Grade**		**Criteria**
High	Method	Disc diffusion/synergy test and PCR<br>Disc diffusion/synergy test and PTA<br>Disc diffusion/synergy test and PFGE<br>Disc diffusion/synergy test and nucleic acid sequencing/mass spectrometry
	Sample size	>15
	Analysis	Data set completely analysed
Medium		Combination of criteria from high and low (e.g., sample size <15 but uses Disc diffusion/synergy test and PCR)
Low	Method	Disc diffusion/synergy test
	Sample size	<15
	Analysis	Data set partly analysed

Supplementary Table 4. Prevalence of antibiotic resistance in commensal *E. coli* isolated from human sources in community settings in low- and middle-income countries.

Antibiotics	Mechanism of inhibition	Study number	Total number of isolates	Number of resistant isolates	Pooled prevalence (%)	Lower bound 95% CI	Upper bound 95% CI	Quality of the evidence (study number)	Reference
Amikacin	Protein synthesis	12	6849	254	4	1	7	High (7), medium (5), low (0)	[3–13]
Amoxicilin	Protein synthesis	1	161	69	43	35	50	High (0), medium (1), low (0)	[14]
Amoxicillin/clavulanic acid	Cell wall synthesis	4	900	222	13	1	32	High (3), medium (1), low (0)	[30,38,40,51]
Ampicillin/sulbactam	Cell wall synthesis	2	427	136	30	26	34	High (1), medium (1), low (0)	[3,8]
Apramycin	Protein synthesis	1	161	0	0	0	1	High (0), medium (1), low (0)	[14]
Azithromycin	Protein synthesis	1	87	10	11	5	19	High (0), medium (1), low (0)	[17]
Aztreonam	Cell wall synthesis	2	258	8	3	1	6	High (2), medium (0), low (0)	[3,7]
Carbenicillin	Cell wall synthesis	1	108	22	20	14	29	High (1), medium (0), low (0)	[7]
Cefazolin	Cell wall synthesis	3	1490	141	8	4	14	High (1), medium (2), low (0)	[3,5,18]
Cefepime	Cell wall synthesis	3	5443	2017	17	1	43	High (1), medium (2), low (0)	[3,4,9]
Cefixime	Cell wall synthesis	1	277	166	60	54	66	High (0), medium (1), low (0)	[8]
Cefoxitin	Cell wall synthesis	6	1371	528	18	0	62	High (4), medium (2), low (0)	[18,30,35,43,50,51]
Ceftazidime	Cell wall synthesis	11	6445	3070	28	12	48	High (7), medium (4), low (0)	[3–5,7,9,13,19–22]
Ceftiofur	Cell wall synthesis	1	7	0	0	0	35	High (1), medium (0), low (0)	[12]
Ceftriaxone	Cell wall synthesis	7	1951	587	25	3	56	High (3), medium (4), low (0)	[30,33,36,41–43,53]
Cefuroxime	Cell wall synthesis	3	535	185	31	8	61	High (2), medium (1), low (0)	[3,7,8]

Cephalothin	Cell wall synthesis	4	404	69	26	22	31	High (1), medium (1), low (0)	[11,15]
Colistin	Cell wall synthesis	2	4764	91	2	1	2	High (1), medium (1), low (0)	[9,13]
Doxycycline	Protein synthesis	1	54	22	41	28	54	High (0), medium (1), low (0)	[23]
Enrofloxacin	Nucleic acid synthesis	1	54	10	18	9	30	High (0), medium (1), low (0)	[23]
Flumequine	Nucleic acid synthesis	1	161	0	0	0	1	High (0), medium (1), low (0)	[14]
Fosfomycin	Cell wall synthesis	2	157	27	25	2	60	High (1), medium (1), low (0)	[21,23]
Gentamicin	Protein synthesis	16	8380	515	9	6	13	High (10), medium (6), low (0)	[4–7,9–13,15,18,20–22,27,28]
Imipenem	Cell wall synthesis	8	5747	317	1	0	4	High (4), medium (4), low (0)	[3–5,7,9,15,20,29]
Kanamycin	Protein synthesis	6	389	48	16	7	27	High (6), medium (0), low (0)	[3,6,10,12,21,28]
Meropenem	Cell wall synthesis	1	4764	434	9	8	10	High (0), medium (1), low (0)	[9]
Neomycin	Protein synthesis	1	161	6	4	1	7	High (0), medium (1), low (0)	[14]
Nitrofurantoin	Nucleic acid synthesis	4	5301	279	3	1	6	High (3), medium (2), low (0)	[7,9–11,13]
Norfloxacin	Nucleic acid synthesis	3	1047	295	36	15	60	High (0), medium (3), low (0)	[4,8,24]
Penicillin	Cell wall synthesis	1	87	41	47	37	57	High (0), medium (1), low (0)	[17]
Piperacillin	Cell wall synthesis	2	850	343	40	27	53	High (0), medium (2), low (0)	[4,11]
Piperacillin/tazobactam	Cell wall synthesis	2	637	13	2	1	7	High (1), medium (1), low (0)	[4,7]
Spectinomycin	Protein synthesis	3	430	176	2	1	4	High (1), medium (1), low (1)	[6,30,31]
Sulfamethoxazole	Folate synthesis	6	5715	2609	53	45	60	High (2), medium (3), low (1)	[9,12,14,23,25,30]

Sulphonamide class	Folate synthesis	4	1399	858	63	56	70	High (3), medium (1), low (0)	[15,31–33]
Tigecycline	Protein synthesis	1	4764	24	0	0	1	High (0), medium (1), low (0)	[9]
Tobramycin	Protein synthesis	1	158	20	8	4	13	High (1), medium (0), low (0)	[10]

a)

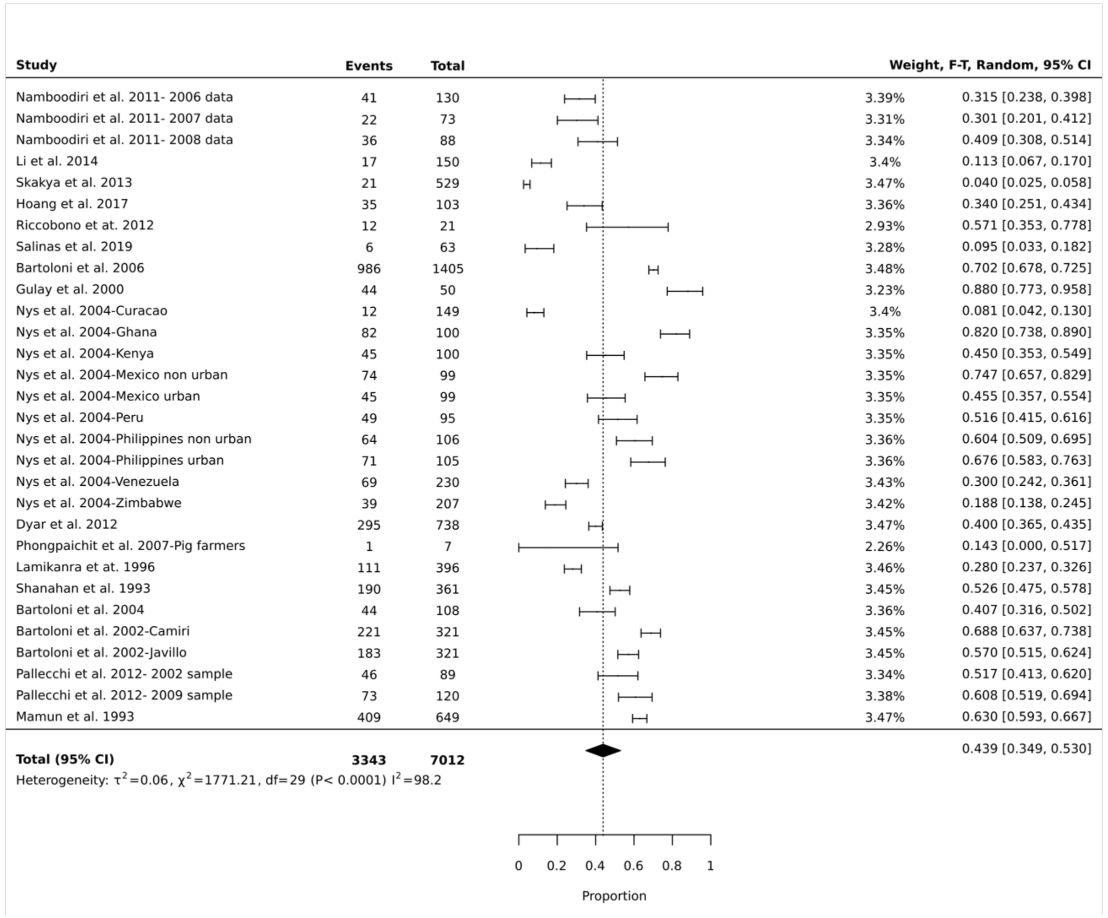
Study Events Total Weight, F-T, Random, 95% CI
Namboodiri et al. 2011- 2006 data 41 130 3.39% 0.315 [0.238, 0.398]
Namboodiri et al. 2011- 2007 data 22 73 3.31% 0.301 [0.201, 0.412]
Namboodiri et al. 2011- 2008 data 36 88 3.34% 0.409 [0.308, 0.514]
Li et al. 2014 17 150 3.4% 0.113 [0.067, 0.170]
Skakya et al. 2013 21 529 3.47% 0.040 [0.025, 0.058]
Hoang et al. 2017 35 103 3.36% 0.340 [0.251, 0.434]
Riccobono et at. 2012 12 21 2.93% 0.571 [0.353, 0.778]
Salinas et al. 2019 6 63 3.28% 0.095 [0.033, 0.182]
Bartoloni et al. 2006 986 1405 3.48% 0.702 [0.678, 0.725]
Gulay et al. 2000 44 50 3.23% 0.880 [0.773, 0.958]
Nys et al. 2004-Curacao 12 149 3.4% 0.081 [0.042, 0.130]
Nys et al. 2004-Ghana 82 100 3.35% 0.820 [0.738, 0.890]
Nys et al. 2004-Kenya 45 100 3.35% 0.450 [0.353, 0.549]
Nys et al. 2004-Mexico non urban 74 99 3.35% 0.747 [0.657, 0.829]
Nys et al. 2004-Mexico urban 45 99 3.35% 0.455 [0.357, 0.554]
Nys et al. 2004-Peru 49 95 3.35% 0.516 [0.415, 0.616]
Nys et al. 2004-Philippines non urban 64 106 3.36% 0.604 [0.509, 0.695]
Nys et al. 2004-Philippines urban 71 105 3.36% 0.676 [0.583, 0.763]
Nys et al. 2004-Venezuela 69 230 3.43% 0.300 [0.242, 0.361]
Nys et al. 2004-Zimbabwe 39 207 3.42% 0.188 [0.138, 0.245]
Dyar et al. 2012 295 738 3.47% 0.400 [0.365, 0.435]
Phongpaichit et al. 2007-Pig farmers 1 7 2.26% 0.143 [0.000, 0.517]
Lamikanra et at. 1996 111 396 3.46% 0.280 [0.237, 0.326]
Shanahan et al. 1993 190 361 3.45% 0.526 [0.475, 0.578]
Bartoloni et al. 2004 44 108 3.36% 0.407 [0.316, 0.502]
Bartoloni et al. 2002-Camiri 221 321 3.45% 0.688 [0.637, 0.738]
Bartoloni et al. 2002-Javillo 183 321 3.45% 0.570 [0.515, 0.624]
Pallecchi et al. 2012- 2002 sample 46 89 3.34% 0.517 [0.413, 0.620]
Pallecchi et al. 2012- 2009 sample 73 120 3.38% 0.608 [0.519, 0.694]
Mamun et al. 1993 409 649 3.47% 0.630 [0.593, 0.667]
Total (95% CI) 3343 7012 0.439 [0.349, 0.530]
Heterogeneity: τ²=0.06, χ²=1771.21, df=29 (P< 0.0001) I²=98.2
0 0.2 0.4 0.6 0.8 1
Proportion

b)

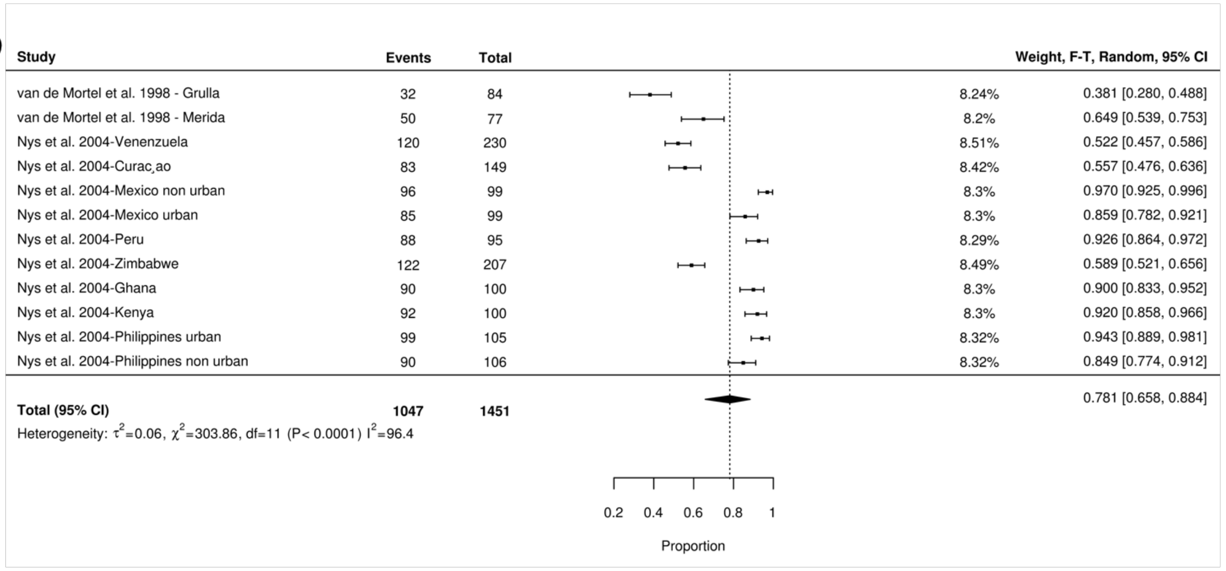
Study Events Total Weight, F-T, Random, 95% CI
van de Mortel et al. 1998 - Grulla 32 84 8.24% 0.381 [0.280, 0.488]
van de Mortel et al. 1998 - Merida 50 77 8.2% 0.649 [0.539, 0.753]
Nys et al. 2004-Venenzuela 120 230 8.51% 0.522 [0.457, 0.586]
Nys et al. 2004-Curac,ao 83 149 8.42% 0.557 [0.476, 0.636]
Nys et al. 2004-Mexico non urban 96 99 8.3% 0.970 [0.925, 0.996]
Nys et al. 2004-Mexico urban 85 99 8.3% 0.859 [0.782, 0.921]
Nys et al. 2004-Peru 88 95 8.29% 0.926 [0.864, 0.972]
Nys et al. 2004-Zimbabwe 122 207 8.49% 0.589 [0.521, 0.656]
Nys et al. 2004-Ghana 90 100 8.3% 0.900 [0.833, 0.952]
Nys et al. 2004-Kenya 92 100 8.3% 0.920 [0.858, 0.966]
Nys et al. 2004-Philippines urban 99 105 8.32% 0.943 [0.889, 0.981]
Nys et al. 2004-Philippines non urban 90 106 8.32% 0.849 [0.774, 0.912]
Total (95% CI) 1047 1451 0.781 [0.658, 0.884]
Heterogeneity: τ²=0.06, χ²=303.86, df=11 (P< 0.0001) I²=96.4
0.2 0.4 0.6 0.8 1
Proportion

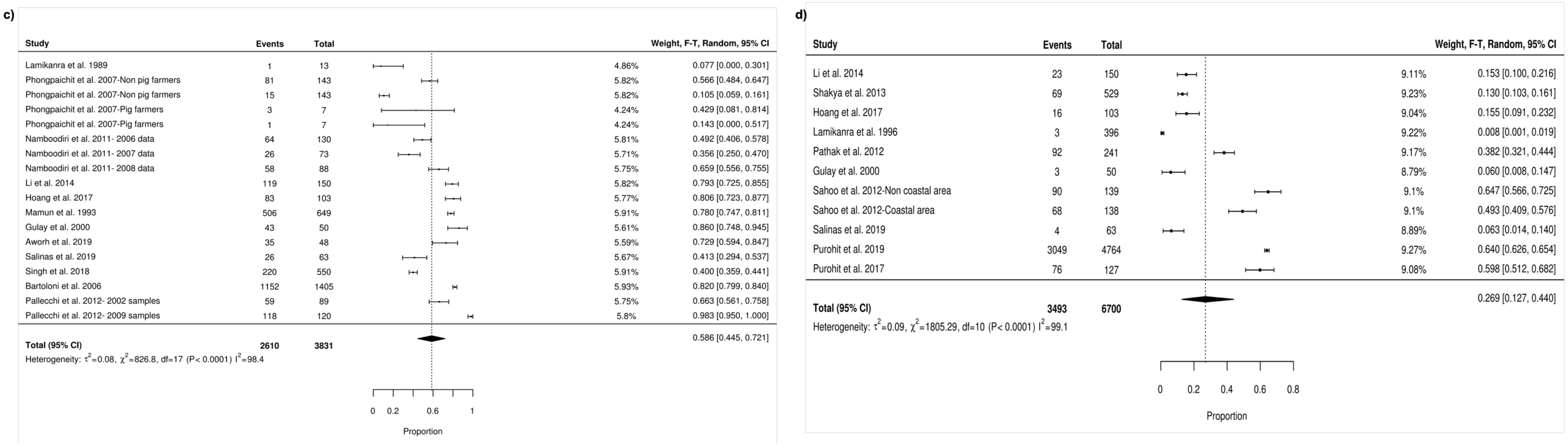

c)
Study
Events
Total
Weight, F-T, Random, 95% CI
Lamikanra et al. 1989 1 13 4.86% 0.077 [0.000, 0.301]
Phongpaichit et al. 2007-Non pig farmers 81 143 5.82% 0.566 [0.484, 0.647]
Phongpaichit et al. 2007-Non pig farmers 15 143 5.82% 0.105 [0.059, 0.161]
Phongpaichit et al. 2007-Pig farmers 3 7 4.24% 0.429 [0.081, 0.814]
Phongpaichit et al. 2007-Pig farmers 1 7 4.24% 0.143 [0.000, 0.517]
Namboodiri et al. 2011- 2006 data 64 130 5.81% 0.492 [0.406, 0.578]
Namboodiri et al. 2011- 2007 data 26 73 5.71% 0.356 [0.250, 0.470]
Namboodiri et al. 2011- 2008 data 58 88 5.75% 0.659 [0.556, 0.755]
Li et al. 2014 119 150 5.82% 0.793 [0.725, 0.855]
Hoang et al. 2017 83 103 5.77% 0.806 [0.723, 0.877]
Mamun et al. 1993 506 649 5.91% 0.780 [0.747, 0.811]
Gulay et al. 2000 43 50 5.61% 0.860 [0.748, 0.945]
Aworh et al. 2019 35 48 5.59% 0.729 [0.594, 0.847]
Salinas et al. 2019 26 63 5.67% 0.413 [0.294, 0.537]
Singh et al. 2018 220 550 5.91% 0.400 [0.359, 0.441]
Bartoloni et al. 2006 1152 1405 5.93% 0.820 [0.799, 0.840]
Pallecchi et al. 2012- 2002 samples 59 89 5.75% 0.663 [0.561, 0.758]
Pallecchi et al. 2012- 2009 samples 118 120 5.8% 0.983 [0.950, 1.000]
Total (95% CI) 2610 3831 0.586 [0.445, 0.721]
Heterogeneity: τ^2=0.08, χ^2=826.8, df=17 (P< 0.0001) I^2=98.4
0 0.2 0.6 1
Proportion
d)
Study
Events
Total
Weight, F-T, Random, 95% CI
Li et al. 2014 23 150 9.11% 0.153 [0.100, 0.216]
Shakya et al. 2013 69 529 9.23% 0.130 [0.103, 0.161]
Hoang et al. 2017 16 103 9.04% 0.155 [0.091, 0.232]
Lamikanra et al. 1996 3 396 9.22% 0.008 [0.001, 0.019]
Pathak et al. 2012 92 241 9.17% 0.382 [0.321, 0.444]
Gulay et al. 2000 3 50 8.79% 0.060 [0.008, 0.147]
Sahoo et al. 2012-Non coastal area 90 139 9.1% 0.647 [0.566, 0.725]
Sahoo et al. 2012-Coastal area 68 138 9.1% 0.493 [0.409, 0.576]
Salinas et al. 2019 4 63 8.89% 0.063 [0.014, 0.140]
Purohit et al. 2019 3049 4764 9.27% 0.640 [0.626, 0.654]
Purohit et al. 2017 76 127 9.08% 0.598 [0.512, 0.682]
Total (95% CI) 3493 6700 0.269 [0.127, 0.440]
Heterogeneity: τ^2=0.09, χ^2=1805.29, df=10 (P< 0.0001) I^2=99.1
0 0.2 0.4 0.6 0.8
Proportion

e)

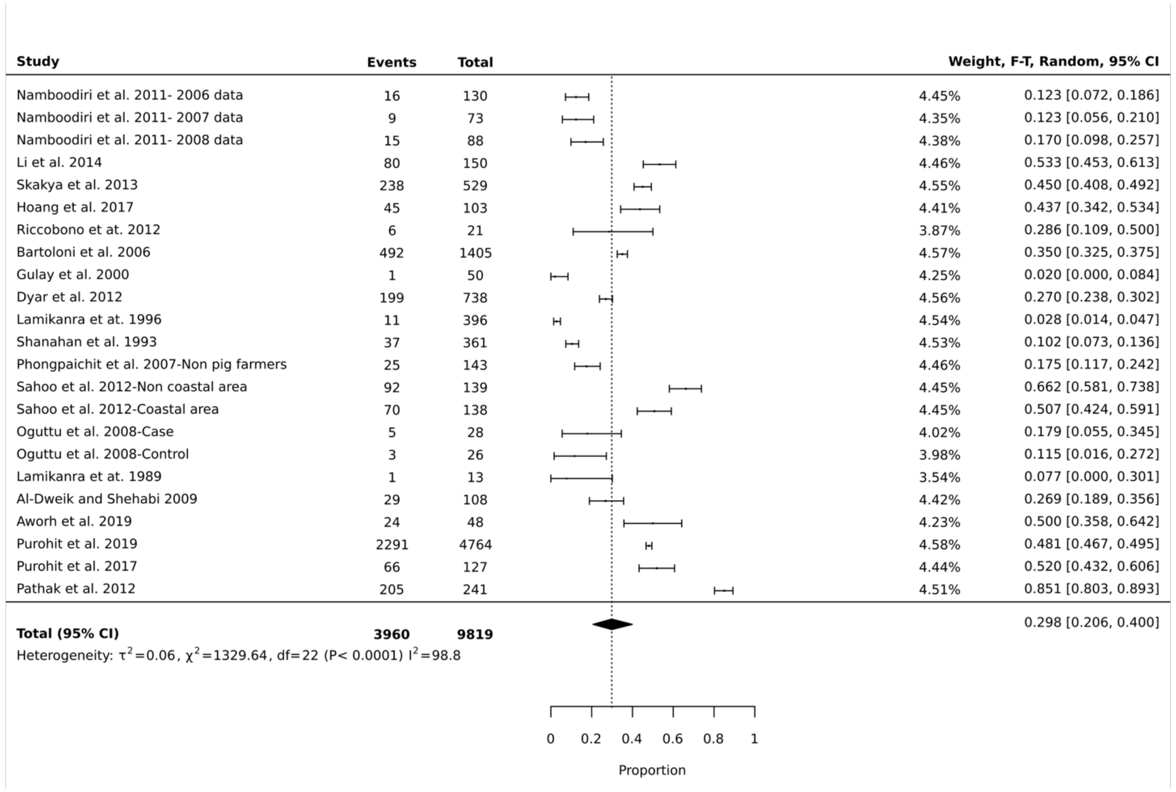

f)

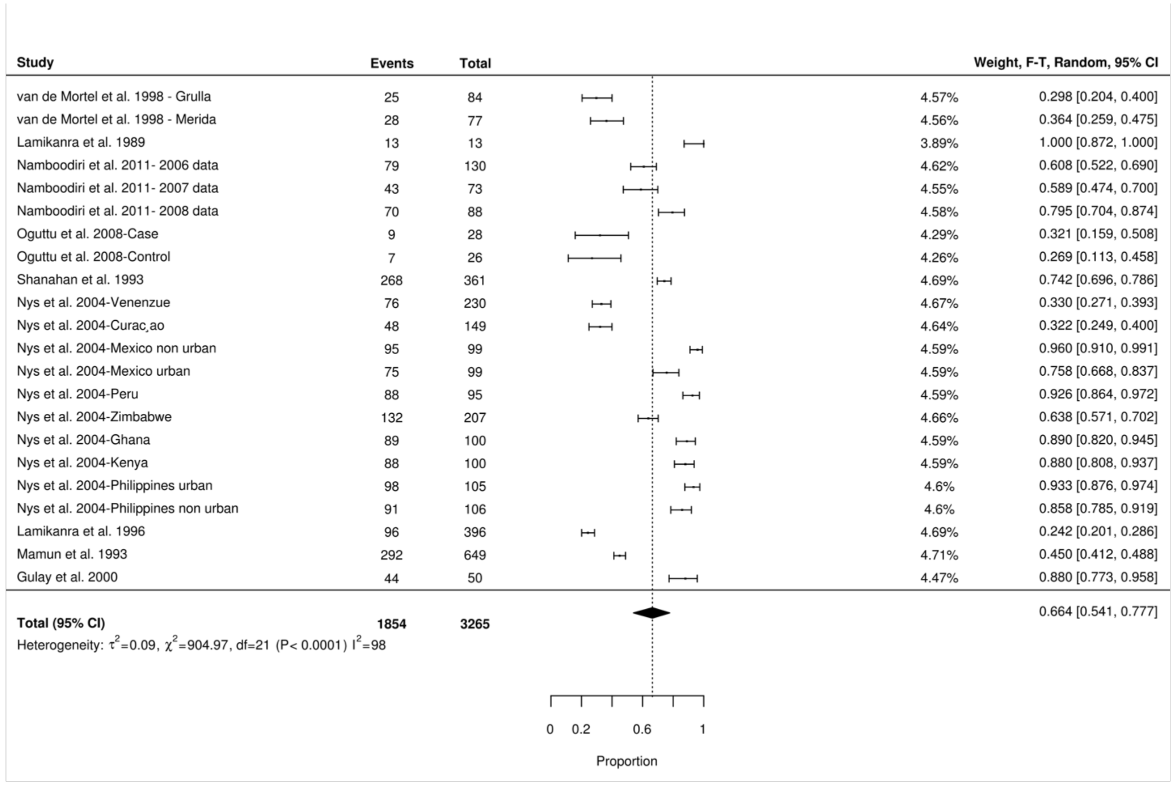

Supplementary Figure 1. Forest plots showing the prevalence of antibiotic resistance in commensal *E. coli* isolated from human sources in community settings in low- and middle-income countries. Protein synthesis inhibitor a) chloramphenicol, b) oxytetracycline, and c) streptomycin. d) Cell wall synthesis inhibitor (cefotaxime), e) Nucleic acid synthesis inhibitor (nalidixic acid), and f) Folate synthesis inhibitor (trimethoprim).

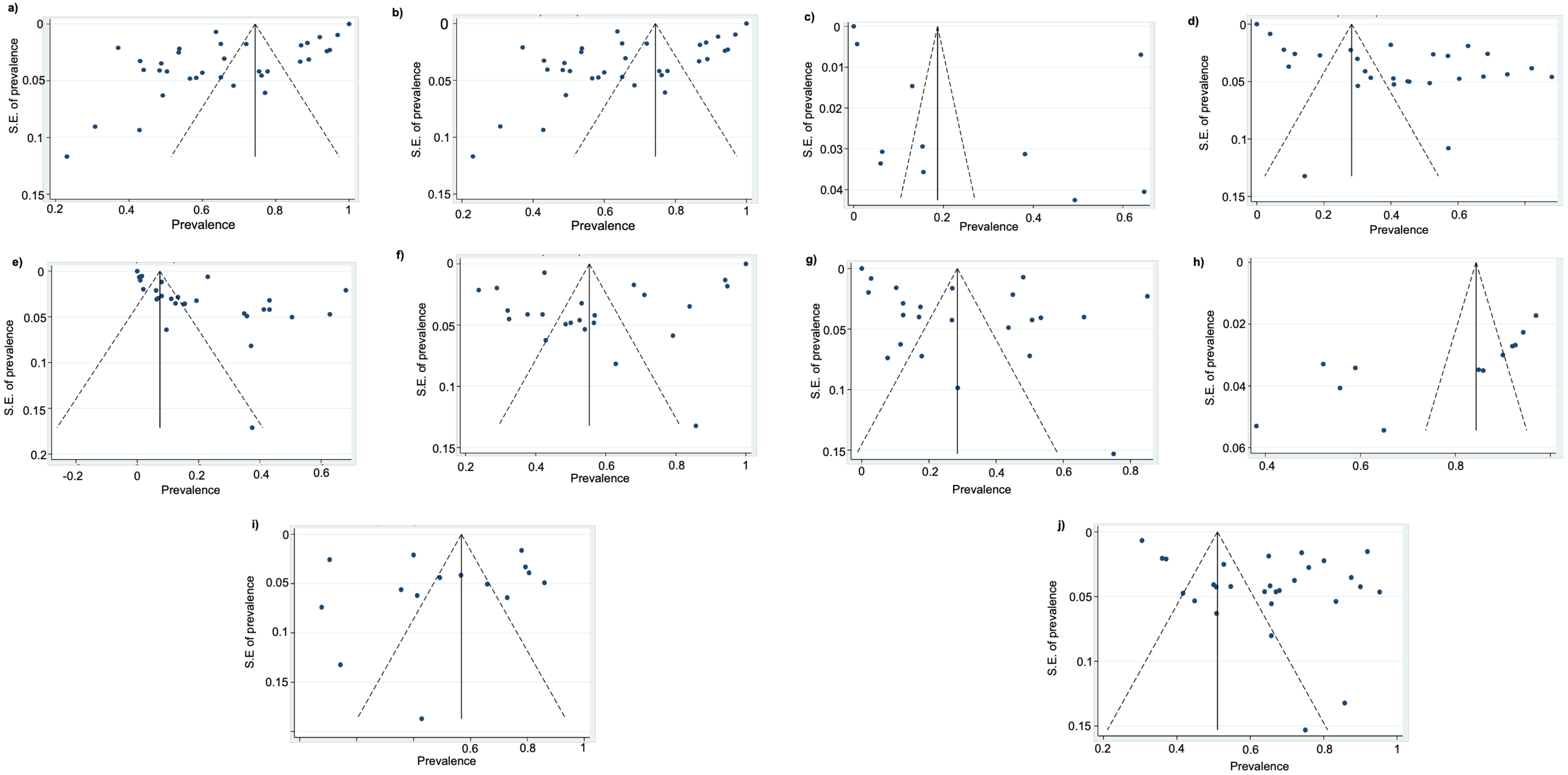

Supplementary Figure 2. Funnel plots for antibiotic resistance in commensal *E. coli* isolated from human sources in community settings in low- and middle-income countries, a) ampicillin, b) cefotaxime, c) chloramphenicol, d) ciprofloxacin, e) co-trimoxazole, f) nalidixic acid, g) oxytetracycline, h) streptomycin, and i) tetracycline.

Supplementary Table 5. Table showing the Egger's regression test results of the top ten antibiotics commonly prescribed in low- and middle-income countries.

**Antibiotic**	**Number of studies**	**Egger bias**	**P value**
All the ten antibiotics	221	9.28	**0.00**
Ampicillin	34	3.50	0.145
Cefotaxime	10	6.97	0.583
Chloramphenicol	27	10.17	**0.002**
Ciprofloxacin	29	6.10	**0.016**
Co-trimoxazole	23	1.13	0.753
Nalidixic acid	22	0.955	0.811
Oxytetracycline	12	14.40	**0.002**
Streptomycin	15	-2.20	0.575
Tetracycline	28	7.71	**0.007**
Trimethoprim	21	-3.06	0.562

Note: Confidence interval - 95%

References

1. Storberg, V. ESBL-producing *Enterobacteriaceae* in Africa – a non-systematic literature review of research published 2008–2012. *Infect. Ecol. Epidemiol.* **4**, 20342 (2014).
2. Hedin, A. & Källestål, C. in *Knowledge-based public health work Part 2, National institute of public health, Stockholm (2004).*
3. Li, B. *et al.* Antimicrobial resistance and integrons of commensal Escherichia coli strains from healthy humans in China. *J. Chemother. Florence Italy* **26**, 190–192 (2014).
4. Shakya, P. *et al.* Antibiotic resistance among Escherichia coli isolates from stool samples of children aged 3 to 14 years from Ujjain, India. *BMC Infect. Dis.* **13**, 477 (2013).
5. Gulay, Z., Bicmen, M., Amyes, S. G. & Yulug, N. Beta-lactamase patterns and betalactam/clavulanic acid resistance in Escherichia coli isolated from fecal samples from healthy volunteers. *J. Chemother. Florence Italy* **12**, 208–215 (2000).
6. Riccobono, E. *et al.* Carriage of antibiotic-resistant Escherichia coli among healthy children and home-raised chickens: a household study in a resource-limited setting. *Microb. Drug Resist. Larchmt. N* **18**, 83–87 (2012).
7. Al-Dweik, M. R. & Shehabi, A. A. Common antimicrobial resistance phenotypes and genotypes of fecal Escherichia coli isolates from a single family over a 6-month period. *Microb. Drug Resist. Larchmt. N* **15**, 103–107 (2009).
8. Sahoo, K. C. *et al.* Geographical variation in antibiotic-resistant Escherichia coli isolates from stool, cow-dung and drinking water. *Int. J. Environ. Res. Public. Health* **9**, 746–759 (2012).
9. Purohit, M. R., Lindahl, L. F., Diwan, V., Marrone, G. & Lundborg, C. S. High levels of drug resistance in commensal E. coli in a cohort of children from rural central India. *Sci. Rep.* **9**, 6682 (2019).
10. Bartoloni, A. *et al.* High prevalence of acquired antimicrobial resistance unrelated to heavy antimicrobial consumption. *J. Infect. Dis.* **189**, 1291–1294 (2004).
11. Bartoloni, A. *et al.* Patterns of antimicrobial use and antimicrobial resistance among healthy children in Bolivia. *Trop. Med. Int. Health* **3**, 116–123 (1998).
12. Phongpaichit, S., Liamthong, S., Mathew, A. G. & Chethanond, U. Prevalence of class 1 integrons in commensal Escherichia coli from pigs and pig farmers in Thailand. *J. Food Prot.* **70**, 292–299 (2007).
13. Purohit, M. *et al.* Antibiotic Resistance in an Indian Rural Community: A 'One-Health' Observational Study on Commensal Coliform from Humans, Animals, and Water. *Int. J. Environ. Res. Public. Health* **14**, 386 (2017).
14. van de Mortel, H. J. *et al.* The prevalence of antibiotic-resistant faecal Escherichia coli in healthy volunteers in Venezuela. *Infection* **26**, 292–297 (1998).
15. Salinas, L. *et al.* Diverse Commensal Escherichia coli Clones and Plasmids Disseminate Antimicrobial Resistance Genes in Domestic Animals and Children in a Semirural Community in Ecuador. *mSphere* **4**, (2019).
16. Qiu, J. *et al.* Molecular and Phenotypic Characteristics of Escherichia coli Isolates from Farmed Minks in Zhucheng, China. *BioMed Res. Int.* **2019**, 3917841 (2019).

17. DeFrancesco, A. S., Tanih, N. F., Samie, A., Guerrant, R. L. & Bessong, P. O. Antibiotic resistance patterns and beta-lactamase identification in Escherichia coli isolated from young children in rural Limpopo Province, South Africa: The MAL-ED cohort. *South Afr. Med. J. Suid-Afr. Tydskr. Vir Geneeskd.* **107**, 205–214 (2017).
18. Nys, S. *et al.* Antibiotic resistance of faecal Escherichia coli from healthy volunteers from eight developing countries. *J. Antimicrob. Chemother.* **54**, 952–955 (2004).
19. Singh, A. K. *et al.* Prevalence of antibiotic resistance in commensal Escherichia coli among the children in rural hill communities of Northeast India. *PloS One* **13**, e0199179 (2018).
20. Chirindze, L. M. *et al.* Faecal colonization of E. coli and Klebsiella spp. producing extended-spectrum beta-lactamases and plasmid-mediated AmpC in Mozambican university students. *BMC Infect. Dis.* **18**, 244 (2018).
21. Hoang, P. H. *et al.* Antimicrobial resistance profiles and molecular characterization of Escherichia coli strains isolated from healthy adults in Ho Chi Minh City, Vietnam. *J. Vet. Med. Sci.* **79**, 479–485 (2017).
22. Newman, M. J. & Seidu, A. Carriage of antimicrobial resistant Escherichia coli in adult intestinal flora. *West Afr. J. Med.* **21**, 48–50 (2002).
23. Oguttu, J. W., Veary, C. M. & Picard, J. A. Antimicrobial drug resistance of Escherichia coli isolated from poultry abattoir workers at risk and broilers on antimicrobials. *J. S. Afr. Vet. Assoc.* **79**, 161–166 (2008).
24. Pathak, A. *et al.* Factors associated with carriage of multi-resistant commensal Escherichia coli among postmenopausal women in Ujjain, India. *Scand. J. Infect. Dis.* **44**, 973–977 (2012).
25. Kalter, H. D. *et al.* Risk factors for antibiotic-resistant Escherichia coli carriage in young children in Peru: community-based cross-sectional prevalence study. *Am. J. Trop. Med. Hyg.* **82**, 879–888 (2010).
26. Thi Quynh Nhi, L. *et al.* Excess body weight and age associated with the carriage of fluoroquinolone and third-generation cephalosporin resistance genes in commensal Escherichia coli from a cohort of urban Vietnamese children. *J. Med. Microbiol.* **67**, 1457–1466 (2018).
27. Shanahan, P. M. *et al.* The prevalence of antimicrobial resistance in human faecal flora in South Africa. *Epidemiol. Infect.* **111**, 221–228 (1993).
28. Bartoloni, A. *et al.* Multidrug-resistant Commensal *Escherichia coli* in Children, Peru and Bolivia. *Emerg. Infect. Dis.* **12**, 907–913 (2006).
29. Aworh, M. K., Kwaga, J., Okolocha, E., Mba, N. & Thakur, S. Prevalence and risk factors for multi-drug resistant Escherichia coli among poultry workers in the Federal Capital Territory, Abuja, Nigeria. *PloS One* **14**, e0225379 (2019).
30. Lamikanra, A., Fayinka, S. T. & Olusanya, O. O. Transfer of low level trimethoprim resistance in faecal isolates obtained from apparently healthy Nigerian students. *FEMS Microbiol. Lett.* **59**, 275–278 (1989).
31. Lamikanra, A., Ako-Nai, A. K. & Ogunniyi, D. A. Transferable antibiotic resistance in Escherichia coli isolated from healthy Nigerian school children. *Int. J. Antimicrob. Agents* **7**, 59–64 (1996).
32. Namboodiri, S. S., Opintan, J. A., Lijek, R. S., Newman, M. J. & Okeke, I. N. Quinolone resistance in Escherichia coli from Accra, Ghana. *BMC Microbiol.* **11**, 44 (2011).
33. Mamun, K. Z., Shears, P. & Hart, C. A. The prevalence and genetics of resistance to commonly used antimicrobial agents in faecal Enterobacteriaceae from children in Bangladesh. *Epidemiol. Infect.* **110**, 447–458 (1993).
